# Obesity Resistance Promotes Mild Contractile Dysfunction Associated with Intracellular Ca^2+^ Handling

**DOI:** 10.5935/abc.20150134

**Published:** 2015-12

**Authors:** Felipe Gonçalves dos Santos de Sá, Ana Paula Lima-Leopoldo, Bruno Barcellos Jacobsen, Artur Junio Togneri Ferron, Wagner Muller Estevam, Dijon Henrique Salomé Campos, Edson Castardeli, Márcia Regina Holanda da Cunha, Antonio Carlos Cicogna, André Soares Leopoldo

**Affiliations:** 1 Centro de Educação Física e Desportos - Departamento de Desportos - Universidade Federal do Espírito Santo, Vitória, ES, Brazil; 2 Departamento de Clínica Médica - Faculdade de Medicina - Universidade Estadual Paulista, Botucatu, São Paulo - Brazil

**Keywords:** Obesity-Resistance, High-Fat Diet, Cardiac Function, Ca^2+^ Handling, Rats

## Abstract

**Background:**

Diet-induced obesity is frequently used to demonstrate cardiac dysfunction. However, some rats, like humans, are susceptible to developing an obesity phenotype, whereas others are resistant to that.

**Objective:**

To evaluate the association between obesity resistance and cardiac function, and the impact of obesity resistance on calcium handling.

**Methods:**

Thirty-day-old male Wistar rats were distributed into two groups, each with 54 animals: control (C; standard diet) and obese (four palatable high-fat diets) for 15 weeks. After the experimental protocol, rats consuming the high-fat diets were classified according to the adiposity index and subdivided into obesity-prone (OP) and obesity-resistant (OR). Nutritional profile, comorbidities, and cardiac remodeling were evaluated. Cardiac function was assessed by papillary muscle evaluation at baseline and after inotropic maneuvers.

**Results:**

The high-fat diets promoted increase in body fat and adiposity index in OP rats compared with C and OR rats. Glucose, lipid, and blood pressure profiles remained unchanged in OR rats. In addition, the total heart weight and the weight of the left and right ventricles in OR rats were lower than those in OP rats, but similar to those in C rats. Baseline cardiac muscle data were similar in all rats, but myocardial responsiveness to a post-rest contraction stimulus was compromised in OP and OR rats compared with C rats.

**Conclusion:**

Obesity resistance promoted specific changes in the contraction phase without changes in the relaxation phase. This mild abnormality may be related to intracellular Ca2+ handling.

## Introduction

Obesity is characterized by an excess of fat mass influenced by genetic and environmental factors^1-3^. This multifactorial disease is an independent risk factor for cardiovascular disorders such as hypertension, arteriosclerosis, and coronary heart disease^1,4^.

Elucidation of the mechanisms involved in obesity-related cardiac dysfunction requires the use of appropriate diet-induced models^4,5-9^. However, it is well known that rats, like humans, show different susceptibilities to the development of diet-induced obesity, so it is possible to identify subgroups developing obesity and others maintaining a lean phenotype despite a high caloric intake This subgroup of rats that do not become obese even when fed a high-fat diet are categorized as obesity-resistant (OR) rats^10-12^. These OR rats exhibit lower body weight (BW) gain and less fat deposits than obesity-prone (OP) rats despite similar energy intake^10,13^. Obesity resistance reflects the ability to accurately sense energy balance and respond to increased energy intake with adaptive responses that counteract a tendency for weight gain^14^.

Few studies have evaluated the correlation between cardiac function and obesity resistance, and the mechanisms by which OR can promote myocardial dysfunction are not well understood. Carroll et al^4^ were unable to identify any cardiac abnormalities in OR animals after 12 weeks. Conversely, Louis et al^8^ showed that OR rats fed a high-fat diet for 17 weeks manifested cardiac dysfunction, reflected by significantly increased isovolumetric relaxation time. Several factors have been proposed as contributors to cardiac dysfunction in obesity models, among them changes in calcium (Ca^2+^) handling^5,9,15^. Nevertheless, it is unclear whether changes in Ca^2+^ handling play a critical role in the development of myocardial dysfunction induced by obesity resistance.

Considering the lack of information regarding cardiac function and the mechanisms underlying the involvement of Ca^2+^ handling in obesity resistance, this study was designed to test the hypothesis that obesity resistance does not promote myocardial dysfunction or impairs Ca^2+^ handling in obesity models.

## Methods

### Animal Models and Experimental Protocol

Thirty-day-old male Wistar rats were randomly distributed into two groups: control (C, n = 54) and obese (Ob, n = 54). The C group was fed a standard diet (RC Focus 1765) and the Ob group was alternately exposed to four palatable high-fat diets (RC Focus 2413, 2414, 2415, and 2416; Agroceres, Rio Claro, Brazil) as previously described^5^. The sample size was based on previous studies performed in our laboratory^6,16,23,29^.

BW was recorded weekly after the start of the experimental protocol. Obesity, determined according to BW gain, began to establish at week 3, as previously demonstrated^16^. At this time point, C and Ob rats were maintained on their respective diets for 15 additional consecutive weeks.

### Animal Care

The animals were maintained in a controlled environment with clean air, 12 hours of light/dark cycles starting at 6 a.m., room temperature maintained at 23 ± 3°C, and relative humidity maintained at 60 ± 5%. All experiments and procedures were performed in accordance with the Guide for the Care and Use of Laboratory Animals published by the National Research Council (1996), and were approved by the Ethics Committee for the Use of Animals (UNESP, Botucatu, SP, Brazil), under number 1036.

### Nutritional Profile

Food consumption, calorie intake (CI), feed efficiency (FE), and BW were recorded weekly as previously described^5^. Fifteen weeks after obesity had developed, the animals were anesthetized with an injection of ketamine (50 mg/kg) and xylazine (0.5 mg/kg). They were then decapitated and thoracotomized, and the epididymal, retroperitoneal and visceral fat depots were dissected and weighed. The adiposity index was calculated with the following formula: (total body fat/final BW) x 100. Body fat was determined from the sum of the individual weight of each fat pad according to the formula: Body fat = epididymal fat + retroperitoneal fat + visceral fat^16^.

### Determination of Obesity and Obesity

A criterion based on the adiposity index was used to determine the occurrence of obesity and obesity resistance according to several authors^4,11,19^. After 15 weeks, rats consuming high-fat diets were ranked based on their adiposity indexes. Thus, in the current study, rats on the high-fat diet exhibiting the greatest adiposity indexes were referred to as OP (n = 35), whereas those exhibiting the lowest adiposity indexes were referred to as OR (n = 19). Rats that failed to present the normal characteristic of the C group while fed with a standard diet were no longer used (n = 15).

### Systolic Blood Pressure (SBP)

One week before the rats were euthanized, tail SBP was measured with a tail plethysmograph. The animals were warmed in a wooden box at 40°C for 4 minutes to induce tail arterial vasodilation. A sensor coupled to an electro-sphygmomanometer attached to a computer was placed in the tail and the SBP was then measured with a specific software (Biopac Systems Inc., CA, USA).

### Glucose Tolerance and Homeostatic Model Assessment of Insulin Resistance (HOMA-IR)

The experiments were performed in the C (n = 34), OP (n = 31), and OR (n = 13) rats after 15 weeks of treatment. After 4-6 hours of fasting, a blood sample was collected from the tip of their tails. The blood glucose level (baseline condition) of each animal was immediately determined using a handheld glucometer (Accu-Chek Advantage; Roche Diagnostics Co., Indianapolis, IN). Subsequently, an injection of glucose solution dissolved in water was administered intraperitoneally**(Sigma-Aldrich®, St Louis, MO, USA), and blood glucose levels were measured after 15, 30, 60, 90, and 120 minutes^20^. The HOMA-IR reflects the degree of insulin resistance and was calculated with the following formula: HOMA-IR = [fasting glucose (mmol/l) X fasting insulin (mU/ml)]/22.5.

### Metabolic Profile

At the end of the experimental period, the animals were fasted for 12-15 hours, then anesthetized with an intramuscular injection of ketamine (50 mg/kg) and xylazine (0.5 mg/kg), and euthanized by decapitation. Blood samples were collected and the serum was separated by centrifugation at 3,000 X g for 15 minutes at 4°C, and stored at -80°C until further analysis. The serum was analyzed for levels of glucose, triglycerides (TG), total cholesterol (T-Chol), high-density lipoprotein cholesterol (HDL), l*ow-density lipoprotein *cholesterol (LDL), insulin, and leptin. Glucose, TG, T-Chol, HDL, and LDL were measured with an automatic enzymatic analyzer system (Biochemical analyzer BS-200, Mindray, China). Leptin and insulin levels were determined by enzyme-linked immunosorbent assay (ELISA) method using commercial kits (Linco Research Inc., St. Louis, MO, USA).

### Post-Death Morphological Analysis

The rats were euthanized by thoracotomy, and the hearts, ventricles, and tibia were separated, dissected, weighed, and measured. Cardiac remodeling was determined by analyzing the weight of the heart and the left (LV) and right (RV) ventricles, and their correlation with the tibial length.

### Isolated Papillary Muscle

To assess the intrinsic contractile and mechanical properties of the heart, the isolated papillary procedure was employed as previously described^5^. This experiment was performed in C (n = 36), OP (n = 35), and OR (n = 18) rats. The papillary muscles were also evaluated under the baseline condition of 2.5 mM Ca^2+^ and after the inotropic maneuvers of increase in extracellular Ca^2+^concentration and post-rest contraction (PRC) as previously described^5^.

### Statistical Analysis

All analyses were performed using the SigmaStat 3.5 software (SYSTAT Software Inc., San Jose, CA, USA). The distribution of the variables was assessed with the Shapiro-Wilk test, and the results were reported as means ± standard deviations. Comparisons between groups were performed using one-way ANOVA for independent samples, and Tukey’s *post hoc* test. Repeated-measures two-way ANOVA was used to evaluate glucose tolerance and myocardial Ca^2+^ handling. The level of significance was determined at 5 % (α = 0.05).

## Results

### General Characteristics of the Experimental Groups

There was no difference in baseline BW among the groups (Table 1). The high-fat diet promoted a substantial increase in body fat and adiposity index in OP rats compared with C and OR rats. Specifically, OP rats had a 86.4% and 78.8% higher body fat content and 66.9% and 60.5% higher adiposity indexes than C and OR rats, respectively. In addition, epididymal, retroperitoneal, and visceral fat pads, as well as final BW were greater in OP rats compared with C and OR rats. Despite the greater amount of energy in the high-fat diet, the calorie intake was similar in both groups due to a reduced food consumption by OP and OR rats in relation to C rats. In addition, FE was higher in the OP group compared with that in the C group. Although FE values were similar in OP and OR rats, this parameter showed a trend towards a lower result in OR when compared with OP rats (p = 0.077).

**Table 1:** Characteristics of the experimental groups

**Variables**	**Groups**		
	**C (n= 39)**	**OP (n= 35)**	**OR (n= 19)**
IBW (g)	150 ± 12	152 ± 11	152 ± 10
FBW (g)	486 ± 34	545 ± 46*#	492 ± 41
Weight gain (g)	336 ± 31	393 ± 44*#	340 ± 40
Epididymal fat (g)	7.33 ± 1.72	12.9 ± 3.7*#	7.31 ± 1.94
Retroperitoneal fat (g)	8.74 ± 2.09	17.8 ± 6.2*#	9.84 ± 2.58
Visceral fat (g)	5.95 ± 1.34	10.6 ± 3.7*#	6.00 ± 1.21
Body fat (g)	22.2 ± 4.1	41.3 ± 12.8*#	23.1 ± 4.3
Adiposity index (%)	4.51 ± 0.73	7.53 ± 1.99*#	4.69 ± 0.67
FC (g/day)	26.6 ± 1.9	22.4 ± 2.8*	20.8 ± 3.0&
CI (kcal/day)	78.4 ± 5.7	81.6 ± 10.1	75.9 ± 10.9
FE (%)	2.40 ± 0.24	2.85 ± 0.50*	2.59 ± 0.48

Values are expressed as mean ± standard deviation; n: Number of animals; C: Control; OP: Obesity prone; OR: Obesity resistant; IBW: Initial body weight; FBW: Final body weight; FC: Food consumption; CI: Calorie intake; FE: Feed efficiency;

* C versus OP; p < 0.05;

& C versus OR; p < 0.05;

# OP versus OR, p < 0.05; One-way ANOVA for independent samples and Tukey's post hoc test.

### Glucose, Insulin, HOMA-IR, and Metabolic Profile

**Figure 1:**
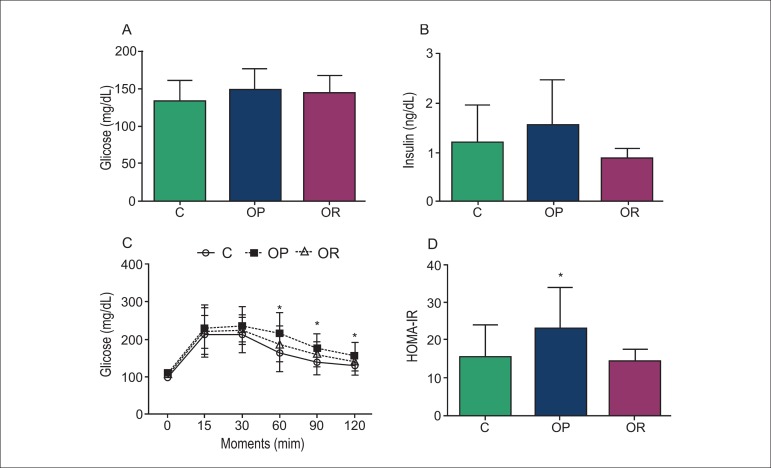
Hormonal profiles and comorbidities; n= number of animals. A: Serum glucose; B: Serum insulin; C: Blood glucose levels following an oral glucose load; D: Homeostasis model assessment of insulin resistance (HOMA-IR) in control (C, white circles; n = 34), obesity-prone (OP, black squares; n = 31) and obesity-resistant (OR, white triangles; n = 13) rats. Values are expressed as mean ± standard deviation. ^*^C versus OP; p < 0.05.

There were no statistical differences in glucose and insulin levels between the groups (Figure 1 - A and B). However, the glucose profile and HOMA-IR index were significantly affected by exposure to obesity (Figure 1 - C and D). The OP rats presented higher levels of glucose at time points 60, 90, and 120 minutes compared with C rats (Figure 1 - C). In addition, there was no statistical difference in the glucose profile between C and OR rats (Figure 1 - C). The area under the curve (AUC) for glucose was higher in OP rats than C rats (Table 2). Moreover, the HOMA-IR index was higher in OP rats than C and OR rats (Figure 1 - D). HDL, LDL, T-Chol and SBP were not significantly different between the groups (Table 2). However, TG levels were higher in OP than C rats. Furthermore, OP rats exhibited higher levels of leptin when compared with C and OR rats (Table 2).

**Table 2:** Systolic blood pressure, and biochemical and hormonal profiles

	**Groups**		
**Variables**	**C (n= 34)**	**OP (n= 31)**	**OR (n= 13)**
AUC (mg/dL/min)	19823±4166	23718 ± 4172*	21713±3887
SBP (mmHg)	128 ± 8	132 ± 11	131 ± 11
Cholesterol (mg/dL)	59.8 ± 14.0	67.2 ± 14.0	63.7 ± 9.1
Triglycerides (mg/dL)	54.6 ± 14.0	74.0 ± 26.2*	62.4 ± 25.7
HDL (mg/dL)	36.2 ± 22.0	38.9 ± 19.5	40.5 ± 19.3
LDL (mg/dL)	25.5 ± 10.1	28.4 ± 13.4	27.6 ± 14.1
Leptin (ng/mL)§	3.45 ± 5.47	6.70 ± 14.30*#	3.25 ± 4.66

Values are expressed as mean ± standard deviation; n: Number of animals; C: Control; OP: Obesity prone; OR: Obesity resistant; AUC: Area under the curve for glucose; SBP: Systolic blood pressure; HDL: High-density lipoprotein cholesterol; LDL: Low-density lipoprotein cholesterol;

§ Values are expressed as median ± semi-interquartile range;

* C versus OP; p < 0.05;

# OP versus OR, p < 0.05; One-wayANOVA for independent samples and Tukey's post hoc test; ^§^Kruskal-Wallis one-way ANOVA and Dunn's post hoc test.

### Morphological Characteristics

**Figure 2:**
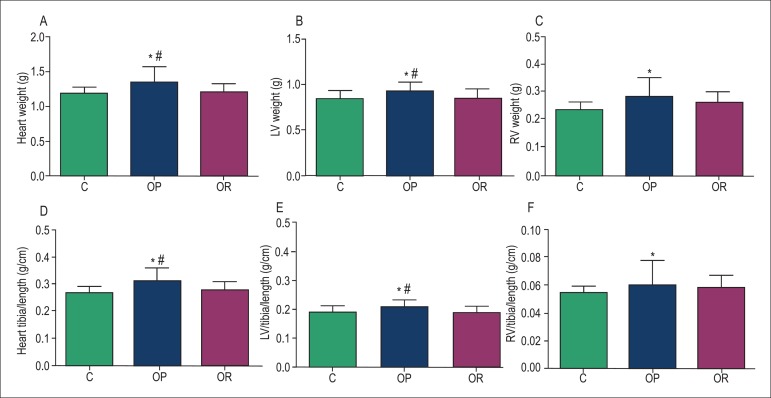
Morphologic profiles of controls (C, n = 39), obesity-prone (OP, n = 35) and obesity-resistant (OR, n = 19) rats; n = Number of animals. A: Heart weight; B: Left ventricular (LV) weight; C: Right ventricular (RV) weight; D: Heart weight/tibial length; E: LV/tibial length: F: RV/tibial length. Values are expressed as mean ± standard deviation. ^*^C versus OP; p < 0.05; #OP versus OR, p < 0.05.

The morphological characteristics of the rats are displayed in Figure 2 (A-F). The absolute heart and LV weights, as well as their correlation with tibial length, were significantly increased in OP rats compared with C and OR rats (Figure 2 - A, B, D and E). Furthermore, OP rats showed greater RV weight and correlation with tibial length than C rats (Figure 2 - C and F). Of note, tibial lengths were similar among the groups.

### Analysis of Myocardial Function and Calcium (Ca^2+^) Handling

**Figure 3:**
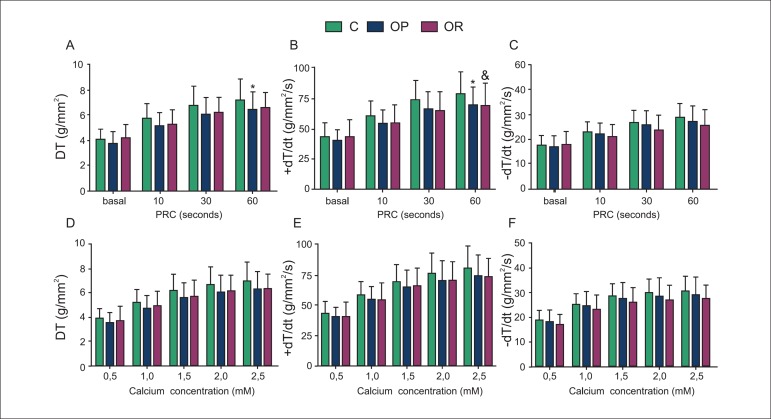
Effects of post-rest contraction (PRC; A, B, and C) and increasing extracellular Ca^2+^ concentration (D, E, and F) in papillary muscles of control (C; n= 36), obesity-prone (OP; n = 35), and obesity-resistant (OR; n= 18) rats. PRC basal: 0.5 mM [Ca^2+^]. Values are expressed as mean ± standard deviation; n = number of animals. DT: maximum developed tension; Peak of the positive (+dT/dt) and negative (-dT/dt) tension derivatives. ^*^C versus OP; p < 0.05; ^&^C versus OR; p < 0.05.

The contraction performance of papillary muscles at baseline conditions (Ca^2+^ of 2.5 mM) was similar for all parameters in the C, OP, and OR groups (Table 3). After 60 seconds of PRC, the maximum developed tension (DT) was lower in OP rats compared with C rats, (C: 7.2 ± 1.7 g/mm^2^ *versus* OP: 6.4 ± 1.4 g/mm^2^; Figure 3 - A). Although the significance of this effect was only observed at 60s, the myocardium from OP rats also exhibited lower values of DT in response to PRC at 30s (C: 6.8 ± 1.5 versus OP: 6.1 ± 1.3, p = 0.056). Although at baseline condition, +dT/dt values were similar between C, OP and OR rats, when subjected to PRC at 60 s, this parameter was reduced in OP and OR rats compared to C rats (Figure 3 - B). In addition, there was a trend towards lower +dT/dt in OP and OR rats during the PRC at 30 seconds, when compared with C rats (p = 0.075 and p = 0.076, respectively), but these values were not significantly different between the groups (Figure 3 - B). Figure 3 (D and E) shows that obesity resistance did not impair DT and +dT/dt after increase in extracellular Ca^2+^ concentration. Furthermore, obesity and obesity resistance failed to elicit any significant effect on the peak of the negative tension derivatives (-dT/dt) at baseline and after maneuvers in the groups (Figure 3 - C and F).

**Table 3:** Baseline data from isolated muscle preparations

**Variables**	**Groups**		
	**C (n= 36)**	**OP (n= 35)**	**OR (n= 18)**
DT (g/mm^2^)	6.77 ± 1.68	6.10 ± 1.68	6.30 ± 1.37
RT (g/mm^2^)	1.22 ± 0.44	1.03 ± 0.38	0.99 ± 0.27
+dT/dt (g/mm^2^/s)	75.9 ± 18.8	70.6 ± 19.2	72.5 ± 16.2
-dT/dt (g/mm^2^/s)	25.3 ± 5.3	24.9 ± 7.0	24.0 ± 5.2
CSA (mm^2^)	1.10 ± 0.25	1.14 ± 0.29	1.10 ± 0.35

Values are expressed as mean ± standard deviation; n: Number of animals; C: Control; OP: Obesity prone; OR: Obesity resistant Baseline condition 2.5 mM [Ca^2+^]; DT Maximum developed tension normalized per cross-sectional area of the papillary muscle; RT: Resting tension normalized per cross-sectional area of the papillary muscle; Peak of the positive (+dT/dt) and negative (-dT/dt) tension derivatives normalized per cross-sectional area of the papillary muscle; CSA: Cross-sectional area; One-way ANOVA for independent samples and Tukey's post hoc test.

## Discussion

Although obesity and overweight are increasingly widespread, some individuals remain resistant to becoming obese^2^. Previous studies have shown that this resistance to obesity may be attributed to changes in nutrition and adiposity patterns^21,22^. Most humans and animals consuming high-fat diets show an increase in BW, with corresponding increase in adiposity levels^23,24^. In comparison, some animals that are fed high-fat diets present less weight gain and adiposity than others that are prone to obesity.

Few studies have evaluated and identified the cardiac characteristics of OR rats^4,8,17^. Still, the occurrence of cardiac dysfunction and its mechanisms remain unknown in this animal model. Interestingly, little information is available on the relationship between obesity resistance, cardiac function, and Ca^2+^ handling. The major finding in the current study was that obesity resistance promotes mild myocardial dysfunction, and this result was related to damage in the contraction phase. We believe that this is the first study to report the role of Ca^2+^ handling in the myocardium of OR rats.

Fat-enriched diets have been used for decades to model obesity and obesity resistance in rodents^17,22,24,25^. Using male rats fed a high-saturated fat diet for 20 weeks, these studies reported that 42.5% and 40% rats were classified as OP and OR, respectively. In addition, Carroll et al^4^ found that 12 weeks of a moderate-fat diet identified 37.5% and 31.25% of OP and OR rats, respectively. The high-fat diet used in the present study was sufficiently intense and long to promote obesity in 64.8% of the rats (OP), whereas 35.2% of the rats did not develop obesity (OR). The literature reveals that obesity resistance is characterized by gain in BW and adiposity at a rate similar to, or lower than that of standard chow-fed rats^12,13,26,27^. The findings of the current study show that OR animals had significantly reduced final BW and fat deposits compared with the OP group, but similar characteristics as those of C rats. In addition, the adiposity index was 60.5% lower in the OR group compared with the OP group. These results are in line with findings of several other studies^17,28^.

Previous studies have shown that obesity resistance can occur due to increased total energy expenditure as well as reduced food intake^11,13,21^. Joo et al^21^ observed increased expression of some thermogenic enzymes and decreased expression of lipogenic enzymes in adipose tissues of OR rats fed a high-fat diet. Obesity resistance also showed suppression of lipogenesis and acceleration of fatty-acid oxidation in visceral fat^13^. The authors suggested that these characteristics are likely to contribute to the anti-obesity phenotype in rats. Moreover, Jackman et al^14^ demonstrated that to maintain body homeostasis, OR animals tend to decrease their food intake and/or increase their energy expenditure.

Many experiments have demonstrated that disorders induced in rats fed a high-fat diet resemble the human comorbidities caused by obesity, such as glucose intolerance, insulin resistance, hypertension, and dyslipidemia^4,18,28-30^. In OR models, there have been controversies regarding the presence of comorbidities^10,31^. In the current study, there were no changes typically associated with obesity in OR rats, since the high-fat diet was not able to promote changes in glucose, lipid, insulin, leptin, or blood pressure profiles. Our data corroborate those of other studies in which elevation of these variables and/or presence of comorbidities were also not identified^4,10,31^. Of note, Carroll et al^4^ found an increase in the HOMA-IR in OR rats compared with C rats.

Morphologic analysis indicated that obesity resistance did not induce cardiac remodeling as seen in human obesity^4^. Instead, OR rats presented lower total heart, and LV and RV weights compared with OP rats. While obesity promoted changes in cardiac structures, such as increase in LV weight (9.0%) and RV weight (21.0%) compared with C rats, OR rats only displayed a slight increase of 8.1% in RV weight, with no significant change in LV weight. Several factors have been implicated in the development of ventricular hypertrophy in obese models, including insulin and leptin^18,32,33^. Our results suggest that leptin and insulin did not increase sufficiently to promote cardiac remodeling in OR rats.

The purpose of the present investigation was to study the changes in LV myocardial performance using the isolated papillary muscle preparation method. Several investigations currently use these maneuvers to identify changes in the contraction and relaxation phases which may not be observed under baseline conditions^9,19,34,35^. Along with a lack of increase in BW or fat in the OR rats, the cardiac function in these animals did not change significantly after exposure to a high-fat diet at baseline conditions. Nevertheless, the myocardial responsiveness to PRC was compromised with specific changes in the contraction phase, but without changes in the relaxation phase. Our data are in disagreement with those of Louis et al^8^ who have shown that OR rats fed a high-fat diet for 17 weeks presented cardiac dysfunction during the relaxation phase. Despite the absence of cardiac dysfunction at baseline conditions, the PRC stimulation provided evidences that the impairment of myocardial contraction seen in OR rats was related to changes in intracellular Ca^2+^ handling. However, there are only a few studies that have reported impaired intracellular Ca^2+^ handling leading to myocardial dysfunction in OR rodents. In cardiac myocytes, Ca^2+^ plays an important role in cardiac performance and physiological processes^15,36^. According to Bögeholz et al^36^, there are three main ways to modulate the contractile function of myofilaments, namely (1) alteration of cytosolic Ca^2+^concentration, (2) mechanical change in pretension, and (3) catecholaminergic stimulation.

A possible explanation for the contraction impairment mediated by +dT/dt in OR rats may be related to β-adrenergic system downregulation^37^, which was not observed in this study. Positive inotropy in response to β-stimulation involves several pathways such as a) phosphorylation of plasma membrane Ca^2+^ channels by protein kinase A increasing Ca^2+^entry into the cell, b) phosphorylation of phospholamban and ryanodine receptor (RyR), increasing Ca^2+^ stores and Ca^2+^ release from the sarcoplasmic reticulum, respectively, and c) increase in actomyosin shortening velocity, which increases crossbridge cycling^37,38^. It has been reported that changes in the β-adrenergic system can reduce L-type Ca^2+^ channels and RyR activity by regulating their phosphorylation status in obesity models^5,23,39,40^.

## Conclusion

In summary, the results from this investigation demonstrate that mild myocardial function changes caused by obesity resistance are related to specific contraction impairment without changes in the relaxation phase. Future studies are necessary to evaluate the damage to intracellular Ca^2+^ handling, as well as the β-adrenergic system in OR rodent models.
